# Recent Advances of Manganese-Based Hybrid Nanomaterials for Cancer Precision Medicine

**DOI:** 10.3389/fonc.2021.707618

**Published:** 2021-10-13

**Authors:** Xiaoman Liu, Pengfei Rong

**Affiliations:** ^1^ Department of Radiology, Third Xiangya Hospital, Central South University, Changsha, China; ^2^ Postdoctoral Research Station of Clinical Medicine, Third Xiangya Hospital, Central South University, Changsha, China; ^3^ College of Traditional Chinese Medicine, Tianjin University of Traditional Chinese Medicine, Tianjin, China

**Keywords:** cancer precision medicine, magnetic resonance imaging, manganese, nanotheranostic, nanomaterials

## Abstract

Cancer precision medicine (CPM) could tailor the best treatment for individual cancer patients, while imaging techniques play important roles in its application. With the characteristics of noninvasion, nonionized, radiation-free, multidimensional imaging function, and real-time monitoring, magnetic resonance imaging (MRI) is an effective way for early tumor detection, and it has become a tower of strength in CPM imaging techniques. Due to linkage with nephrogenic systemic fibrosis (NSF), gadolinium (Gd)-based contrast agent (CA), which was long used in MRI, has been restricted by the Food and Drug Administration (FDA). In this review, we would like to introduce the manganese (Mn)-based CAs that could significantly increase the safety of MRI CAs by realizing more superior performance and functions simultaneously in the diagnosis and treatment of tumors. Also, recent advances in Mn-based hybrid nanomaterials for CPM are summarized and discussed.

## Introduction

Cancer precision medicine (CPM), evolved with the development of novel nanoparticles (NPs) for cancer diagnosis and treatment, could tailor the best treatment for individual cancer patients. Nowadays, CPM has become popular in clinical and bioscience worldwide, with the conventionally used cancer therapies (e.g., chemotherapy, radiotherapy, and surgery) suffering from lower therapeutic efficiency and ineluctable side effects (1–4).

With a large number of nanomaterial-based new cancer therapies being emerged [e.g., photothermal therapy (PTT)/photodynamic therapy (PDT), sonodynamic therapy (SDT), magnetic hyperthermia therapy, etc.], CPM includes an extensive range of cancer management, such as cancer screening and monitoring, drug selection/prediction, and personalized immunotherapy (2, 5–8). CPM relies heavily on imaging methods, including computed tomography (CT), magnetic resonance imaging (MRI), positron emission tomography (PET), and optical imaging (OI), to provide distinct and precise pathological features for patients.

Owing to superb soft tissue imaging contrast, high spatial resolution, multidimensional imaging, and absence of ionizing radiation, MRI becomes increasingly available for early detection of tumors with gadolinium (Gd)-based contrast agents (CAs) most frequently used (9, 10). Unfortunately, Gd-based CA is in restricted use by the Food and Drug Administration (FDA) due to possibly Gd-based CA-linked medical conditions known as nephrogenic systemic fibrosis (NSF), chronic kidney disease (CKD), and severe complexities, which led to new concerns on the safety of Gd as MRI CAs clinically (11–14).

To increase the safety of MRI CAs, manganese (Mn) ion (Mn^2+^), a non-lanthanide metal, a necessary element in cell biology, and the earliest reported CAs used for enhancing T1-weighted MRI, became an optimal choice due to its paramagnetic nature, low toxicity, and high biosafety (15).

Various Mn-based nanomaterials, such as MnCl_2_, Mn chelates, and MnO nanoparticles, have been utilized for cancer diagnosis with great biocompatibility (15–18). Multiple Mn-based nanostructures, such as nanosheets, hollows, nanocages, and nanobubbles, could act as reservoirs for efficient drug delivery (19–22). Additionally, Mn-based hybrid nanomaterials could be adaptable and responsive to both endogenous compounds in the inner tumor microenvironment (TME) (23) and external environmental stimuli, such as acidity, glutathione, temperature, pH, enzyme, light, redox, and chemical signals. Due to those characteristics, Mn-based hybrid nanomaterials could realize demanded discharge of cargo molecular for imaging-guided cancer therapy, thus minifying additional damage in normal tissues (24, 25).

To sum up, the paramagnetism and Fenton-like property of Mn^2+^ have made Mn-based hybrid nanoparticles with multiple effects, including great performance in MRI, drug delivery, and imaging-guided therapy theranostic systems to integrate diagnosis and treatment into a nanoplatform. Mn-based hybrid nanomaterials have brought a new dawn to the treatment of tumors (26).

In this review, we aimed to provide an overview of recent advances in a possible workflow of Mn-based hybrid nanomaterials used for CPM by reviewing recent emerging techniques and treatments that have been used or will be potentially used. The Mn-based hybrid nanomaterials as imaging agents, carriers for drug delivery, and theranostic agents are summarized in sections *Manganese-Based Hybrid Nanomaterials as Imaging Agents*, *Manganese-Based Hybrid Nanomaterials as Carriers for Drug Delivery*, and *Manganese-Based Hybrid Nanomaterials as Theranostic Agents*, respectively. We will discuss how Mn-based hybrid nanomaterials can be used as CAs for detecting and monitoring cancer progression; how they act as chemotherapeutic drug carriers to increase therapeutic index; and how they can function as theranostic agents in imaging-guided PTT, PDT, SDT, and radiation therapy, etc. Here, we highlight the Mn-based hybrid nanomaterials as theranostic agents, and such an imaging-guided nanotheranostic platform would help to develop optimized and individualized regimens in light of patient’s response and offer an opportunity to develop CPM. The progress and perspective are summarized in section *Perspective*.

## Manganese-Based Hybrid Nanomaterials as Imaging Agents

**Figure 1:**
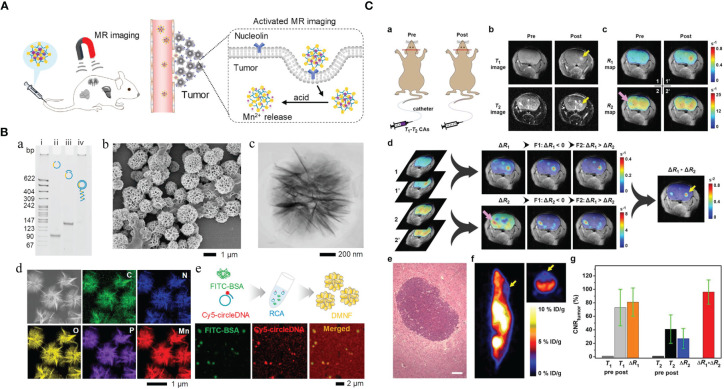
Manganese (Mn)-based hybrid nanomaterials as imaging agents and their application in tumor. **(A)** Diagram of the enhanced MRI of DNA-Mn-based nanoflower (DMNF)-treated tumor-bearing mice (27). **(B)** Preparation and characterization of DMNF imaging agents (27). **(C)** Representative T1- and T2-weighted images of mouse brain at pre- and post-contrast points. T1-T2 dual-modal MRI in brain tumor model through the synthesized MRI contrast agents, NOTA conjugated NEB chelating with Mn^2+^ (Mn-NEB) and BSA (Mn-NEB+BSA) (28). DMNF, DNA-Mn-based nanoflower; NOTA, N, N’, N’’-triacetic acid.

The noninvasive, nonionized, and radiation-free characteristics make MRI one of the most extensively utilized clinical imaging tools. However, conventional signal intensity-based MRI is still limited to its semiquantitative nature, which is susceptible to many factors. Recently, various Mn-based hybrid nanomaterials could increase T1-weighted MRI effect even in acid environment with good biocompatibility or multimodal imaging free from the effects of various conditions in the TME (14, 27, 28). The Mn-based hybrid nanomaterials as imaging agents are summarized in **Table 1**, with the schematic diagram and examples of imaging effect shown in **Figure 1**.

**Table 1:** Manganese-based hybrid nanomaterials as imaging agents.

Agent name	Description	Tumor model	Research group and reference
Mn-NEB+BSA	As dual-modal MRI contrast agents, Mn-NEB+BSA could greatly eliminate suspicious artifacts and false-positive signals in mouse brain imaging.	U87MG tumor-bearing athymic nude mice	Jinhao Gao and Xiaoyuan Chen’s group (28)
DMNF	DMNF showed high tumor-specific MRI with enhanced T1-weighted imaging effect, which was attributed to the synergistic effect of active targeting of AS1411 aptamer and acid-activated release of Mn^2+^ promoting the MR signal enhancement.	MCF-7 tumor-bearing BALB/c nude mice	Dayong Yang’s group (27)
HMS	Hollow manganese silicate (HMS) nanoparticles could release Mn^2+^ in physiological acidic condition as a liver-specific MR contrast agent in hepatic tumor models.	HCC, NEC, and ADC tumor-bearing nude mice	Won Jae Lee and In Su Lee’s group (14)

Mn, manganese; NEB, 1,4,7-triazacyclononane-N, N’, N’’-triacetic acid conjugated truncated Evans blue; BSA, bovine serum albumin; DMNF, DNA-Mn-based nanoflower; HMS, hollow manganese silicate.

T1-T2 dual-modal CAs could enable both T1 bright and T2 dark contrasts. Zhao et al. (27) prepared the multifunctional DNA-Mn-based nanoflower (DMNF), showing enhanced T1-weighted MRI effect even in acid environment and high spatial resolution imaging of kidneys and liver. What is worth mentioning is that Zhou et al. (28) made a 1,4,7-triazacyclononane-N,N’,N’’-triacetic acid-conjugated truncated Evans blue (NEB), and after chelating with Mn (MnNEB) and bovine serum albumin (Mn-NEB+BSA), it could be used as novel T1-T2 dual-modal MRI CA. This study opens a new avenue for contrast-enhanced MRI diagnosis, and it also shows extraordinary promise for CPM (28).

## Manganese-Based Hybrid Nanomaterials as Carriers for Drug Delivery

Nanotechnology acts a great role in drug delivery to help revolutionize CPM. Mn-based hybrid nanomaterials, such as nanosheets, hollow mesoporous nanoshells, and nanocubes, have a high surface-to-volume ratio fit for drug delivery and could produce Mn^2+^ for MRI (20). Currently fabricated composite nanoparticles used for drug delivery include the nanoparticle for the carrier and chemotherapeutic drug for cancer {e.g., doxorubicin [DOX], paclitaxel [PTX], methotrexate [MTX], arsenic trioxide [ATO], cisplatin [cis-diamminedichloroplatinum (CDDP)], etc.} or non-tumor-specific drugs (e.g., hydroxychloroquine, verteporfin, 5-fluorouracil, osteopontin siRNA, etc.) that is either adsorbed, dissolved, or dispersed throughout the nanoparticle complex or covalently attached to the surface of nanoparticles (5). Also, they hold great potential to simultaneously codeliver more drugs in combination therapy. The delivery of non-cytotoxic prodrugs to cancer cells is one of the newer applications (29).

Furthermore, drugs can be formulated at a nanoscale level to increase its therapeutic efficiency. Nanoscale drug delivery systems (nano-DDSs) have already been proposed as a promising way to realize tumor-specific treatment by being adaptable and responsive to many endogenous substances and external stimuli, such as acidity, overexpressed hydrogen peroxide (23), pH, enzyme, light, temperature, and magnetic field.

**Figure 2:**
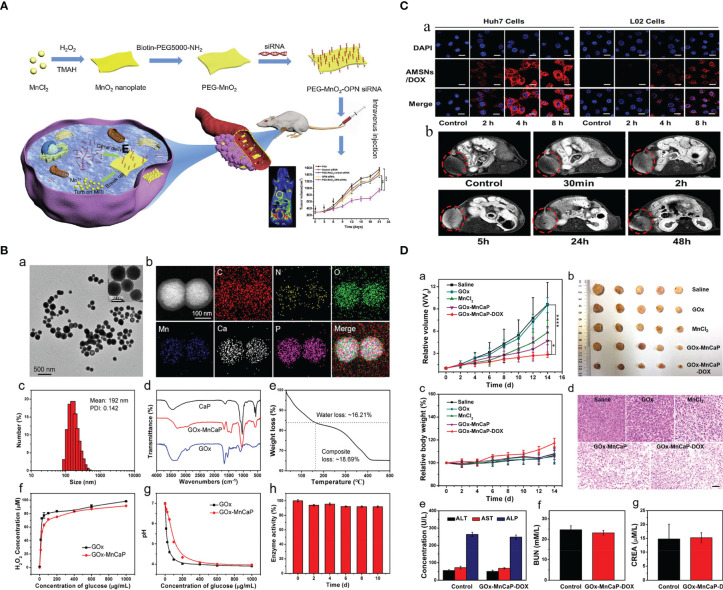
Mn-based hybrid nanomaterials as carriers for drug delivery and their application in tumor. **(A)** Schematic illustration of PEG-MnO_2_-OPN siRNA (20). **(B)** Preparation and characterization of GOx-MnCaP-DOX, glucose oxidase (GOx) with manganese-doped calcium phosphate (MnCaP), and doxorubicin (DOX) (39). **(C)** Tumor cell-selective uptake analysis by confocal laser scanning microscope and *in vivo* tumor homing behavior evaluation by T1-weighted MRI of arginine-rich manganese silicate nanobubbles loading DOX (AMSNs/DOX) (49). **(D)** The *in vivo* antitumor efficacy of GOx-MnCaP-DOX on the 4T1 tumor-bearing mouse model (39). OPN, Osteopontin; GOx, Glucose; MnCaP, manganese-doped calcium phosphate; DOX, doxorubicin; AMSNs, Arginine-rich manganese silicate nanobullles.

Hence, numerous smart hybrid nanomaterials with one or dual stimuli-responsive (e.g., lower pH, hypoxia, tumor-specific enzymes such as glutathione, etc.) drug-releasing and one or dual-mode diagnostic imaging functions (particularly MRI) have been developed to realize improved therapeutic specificity and efficacy (12, 13, 19, 21, 22, 24, 25, 30–48). The Mn-based hybrid nanomaterials as carriers for drug delivery are summarized in **Table 2**, with examples of the schematic diagram for drug delivery system, characterization analysis, and curative effect shown in **Figure 2**. It is worth noting that redox-sensitive Mn-SS (disulfide)/DOX@PDA (polydopamine)-PEG polymers (NCPs) designed by Zhao et al. (30) served as a T1 CA under MRI and showed a glutathione (GSH)-responsive release of DOX. Huang et al. (9) fabricated theranostic nanocomposites Mn-porphyrin&Fe_3_O_4_@SiO_2_@PAA-cRGD and effectively used them in T1- and T2-weighted MRI and pH-responsive drug release. Wang et al. (49) reported the one-pot synthesis of biocompatible arginine-rich Mn silicate nanobubbles (AMSNs) with high tumor killing activity *via* the glutathione-dependent peroxidases 4 (GPX4)-mediated ferroptosis pathway. Such imaging-guided drug-carrying platforms would therefore tremendously promote the development of CPM.

**Table 2:** Manganese-based hybrid nanomaterials as carriers for drug delivery.

Delivered molecules	Agent name	Description	Tumor model	Research group and reference
DOX	HMnO_2_ nanoshells	Hollow mesoporous MnO_2_ (HMnO_2_) nanoshells with DOX loaded could be used for tumor-specific therapy in pH-responsive MRI.	4T1 tumor-bearing Balb/c mice	Zhuang Liu’s group (24)
DOX	Mn-SS/DOX@PDA-PEG NCPs	Redox-sensitive Mn-SS (disulfide)/DOX@PDA (polydopamine)-PEG polymers (NCPs) for T1-contrast MRI and glutathione (GSH)-responsive release of DOX	4T1 tumor-bearing mice	Zili Ge and Zhuang Liu’s group (30)
DOX	MnO_2_-PEG-FA/DOX nanosheets	A redox/pH dual responsive nanotheranostic platform, MnO_2_-PEG-FA/DOX nanosheets through MnO_2_ nanosheets combined with FA and DOX for MRI and chemotherapy	S180 tumor-nearing mice	Zhenzhong Zhang and Yun Zhang’s group (35)
DOX	DOX-GOx-MnCaP NPs	A pH-responsive DOX-loaded glucose oxidase (GOx) with MnCaP spherical nanoparticles for MRI and cascade reaction-enhanced cooperative cancer treatment	4T1 tumor-bearing mice	Peng Huang’s group (39)
DOX	BMDN	MnO_2_/DOX-loaded albumin nanoparticles (BMDN) for MRI and simultaneous chemotherapy	MCF-7/ADR tumor-bearing mice	Huabing Chen and Hu-Lin Jiang’s group (31)
DOX	USMO@MSNs	USMO@MSNs loading DOX for pH-switching MRI and chemotherapy	HSC3 tumor-bearing nude mice	Renfei Wang and Duohong Zou (34)
DOX	Hollow MCO NPs	Hollow manganese/cobalt oxide nanoparticles (MCO-70 NPs) with a tunable size for GSH-responsive dual T1/T2-weighted MRI reporting drug release of DOX	U87MG tumor-bearing nude mice	Zhiping Wan, Junqing Hu, and Yijing Liu’s groups (22)
PTX	W-PTX-MNPs-PPR	Three shaped Mn-Zn ferrite (Mn_0.63_Zn_0.37_Fe_2_O_4_) MNPs for more efficient dual-mode MRI/fluorescence imaging-guided drug delivery	4T1 tumor-bearing mice	Ning Gu and Fei Xiong’s group (41)
MTX	MTX-Mn@PEG NCPs	A chelating agent free, stoichiometry, and pH-responsive NCPs for MRI-guided MTX delivery	HeLa tumor-nearing BALB/c nude mice	Youfu Wang, Dawei Li and Xinyuan Zhu’s group (38)
ATO	[Mn(HAsO_3_)]n@SiO_2_	A pH-sensitive multifunctional trioxide (ATO) drug delivery system (MDDS) through hollow silica nanoparticles loading water-insoluble manganese-arsenite complexes (MnAsOx@SiO_2_) and ATO for real-time monitoring of ATO release by activatable MRI	H22 tumor-nearing BALB/c mice	Jinhao Gao’s group (36)
CDDP	MnO_2_/HA/CDDP nanosheets	MnO_2_/HA/CDDP nanosheets (MnO_2_ nanosheets functionalized by HA, with CDDP absorbed) for pH-responsive MRI and delivering CDDP	A549 tumor-bearing mice	Zhenzhong Zhang and Yun Zhang’s group (37)
HCQ	HA-Mn_2_O_3_/HCQ	TME-responsive drug release and tumor targeting drug carriers-Hollow mesoporous Mn_2_O_3_ NPs conjugated with hyaluronic acid (HA) loading hydroxychloroquine (HCQ, traditional autophagy inhibitor) into the hollow core, for MRI-guided *in situ* autophagy inhibition	4T1 tumor-bearing BALB/c mice	Lin Hou and Zhenzhong Zhang’s group (21)
BPD	MnO_2_/BPD NPs	MnO_2_/BPD nanocomposites for vessel embolization therapy with MR, PA, and FL multimodal imaging as a predictor	Hep-G2 tumor-bearing BALB/c mice	Meng Niu, Ke Xu and Jie Tian’s group (19)
OPN siRNA	PEG-MnO_2_-OPN siRNA	PEG-modified MnO_2_ nanosheets carrying osteopontin (OPN) siRNA for GSH-responsive MRI-guided gene delivery	786-O tumor-bearing mice	Kai Xua and Jingjing Li’s group (20)
5-Fu	Mn-ZIF-8/5-Fu NPs	A pH-responsive bimetallic zeolitic imidazolate framework (Mn-ZIF-8) loading 5-fluorouracil showing diagnostic (MRI) and improved therapeutic applications in U87-MG tumor-bearing mice	U87-MG tumor-bearing Balb/c nude mice	Jianhua Wang, Anwen Shao, and Jianmin Zhang’s group (40)

HMnO_2_, hollow mesoporous MnO_2_; DOX, doxorubicin; MRI, magnetic resonance imaging; PDA, polydopamine; SS, disulfide; GSH, glutathione; GOx, glucose oxidase; MnCaP, manganese-doped calcium phosphate; BMDN, BSA-MnO_2_-DOX nanoparticles; USMO@MSNs, Ultrasmall manganese oxide-capped mesoporous silica nanoparticles; MCO-70 NPs, Hollow manganese/cobalt oxide nanoparticles with an average size of 70 nm; MTX, methotrexate; MDDS, multifunctional drug delivery system; MnAsOx@SiO_2_, hollow silica nanoparticles loading water-insoluble manganese-arsenite complexes; HA, hyaluronic acid; CDDP, cis diamminedichloroplatinum; TME, tumor microenvironment; HA, hyaluronic acid; HCQ, hydroxychloroquine; BPD, benzoporphyrin derivative; Mn_2_O_3_, manganese trioxide; OPN, osteopontin; ZIF, zeolitic imidazolate framework.

## Manganese-Based Hybrid Nanomaterials as Theranostic Agents

**Figure 3:**
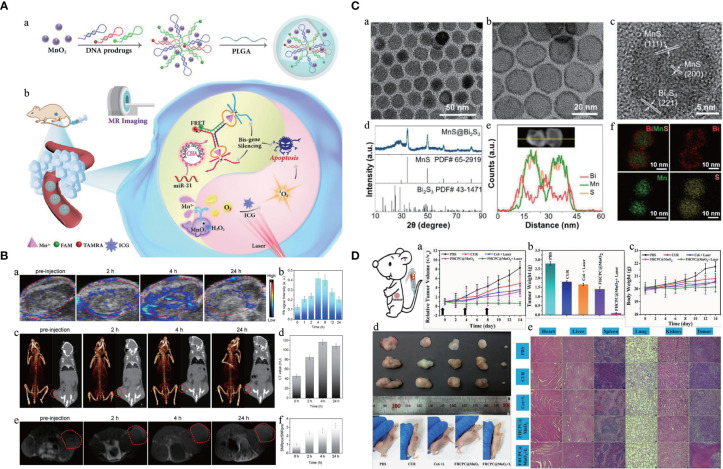
Manganese (Mn)-based hybrid nanomaterials as theranostic agents and their application in tumor. **(A)** Schematic illustration of multifunctional nanocapsule and the systemic delivery of the self-sufficient theranostic nanoplatform (64). **(B)** Preparation and characterization of a core–shell MnS@Bi_2_S_3_-PEG nanostructure theranostic agents (70). **(C)** *In vivo* multimodal imaging (PA images, CT images, and MR images with the red circles mark the tumors) and corresponding signal analysis of tumor-bearing mice before and after intravenous injection of the monolayer bi-anchored Mn boride nanosheets (MBBN) (71). **(D)** *In vivo* therapeutic evaluation of FHCPC@MnO_2_ nanoflowers (polyphosphazene coated onto Fe_3_O_4_ nanoclusters, with MnO_2_ nanosheets as outer shell). Scale bar = 75 μm (72). PA, photoacoustic; FHCPC, coating multifunctional polyphosphazenes onto Fe_3_O_4_ nanoclusters and then growing manganese oxide nanosheets as outer shell; MBBN, Bi-anchored manganese boride nanosheets.

Many efforts have been made for cancer therapy, and the idea of theranostics could help develop a smart nanoparticle to integrate cancer diagnosis, drug delivery, and therapy monitoring simultaneously in a system (50). The intelligent stimuli-responsive manner could offer an efficient strategy for CPM by employing the unique features of TME or clinical external irradiations. With the improvement of polymerization and emulsifying techniques, nanoparticles could be made with hydrophilic and hydrophobic facets to load with different active materials for theranostics. The Mn-based hybrid nanomaterials as imaging agents and carriers for drug delivery have been summarized and discussed in this section, and the Mn-based hybrid nanomaterials as theranostic agents are summarized in **Table 3**, with the schematic diagram and examples shown in **Figure 3**.

**Table 3:** Manganese-based hybrid nanomaterials as theranostic agents.

Therapy	Agent name	Description	Tumor model	Research group and reference
PPT	Au@Mn_3_O_4_ magneto- plasmonic nanoflowers	With great potential in T1-weighted MRI and photothermal therapy (PPT) *in vitro* and *in vivo*	4T1 tumor-bearing mice	Aiguo Wu’s group (11)
PPT	MONPs-BSA-EDTA	For multifunctional imaging-guided PPT	HCT116 tumor-bearing mice	Jing Zhou’s group (51)
PPT	Cu_2_MnS_2_ NPs	For MRI/MSOT dual-modal imaging-guided PTT of cancer in the NIR-II window	S180 tumor-bearing mice	Chunhua Lu and Huanghao Yang’s group (52)
PPT	MNP-Mn	A multifunctional nanoplatform for MR/PA dual-modal imaging-guided PTT	Hep-2 tumor-bearing mice	Ruiping Zhang’s group (53)
PPT	Mn^2+^-doped PB nanocubes	Mn^2+^-doped PB (PB : Mn) nanocubes for MRI-guided PTT with enhanced performance	4T1 tumor-bearing Balb/c mice	Liang Cheng and Zhuang Liu’s group (48)
PDT	FMCNPs	Amphiphilic amino acid-coordinated ionic manganese simultaneous encapsulation of chlorin e6 (FMCNPs) for MRI-guided PDT	MCF7 tumor-bearing mice	Xia Xin, Shiling Yuan, and Xuehai Yan’s group (54)
PDT	MnIO-dBSA	Manganese-doped iron oxide nanoparticles modified with denatured bovine serum albumin (MnIO-dBSA) composites for efficient tumor MRI and PDT	4T1 tumor-bearing mice	Zhijun Zhang’s group (10)
PDT	IHM	By encapsulating a MnO_2_ NP in an ICG-modified hyaluronic acid nanoparticle (HANP) for fluorescent and PA imaging-guided tumor PDT	SCC7 tumor-bearing mice	Guoqing Zhao, Qingjie Ma, and Lei Zhu’s group (55)
PDT	P-AgNCs-MnO_2_	A novel multifunctional DNA-templated silver nanoclusters/porphyrin/MnO_2_ theranostic nanoplatform for non-labeled fluorescence images of Zn^2+^ and PDT	MCF-7 tumor-bearing mice	Daoquan Tang and Fenglei Gao’s group (56)
SDT	DVDMS-Mn-LPs	Encapsulation of DVDMS chelating with Mn into nanoliposomes for integrating imaging and therapy into a single nano-platform	U87 tumor-bearing mice	Fei Yan’s group (57)
SDT	Mn-MOF	A nanosensitizer to self-supply O_2_ and decrease GSH for enhanced SDT and ferroptosis	H22 and 4T1 tumor-bearing mice	Xiangliang Yang and Lu Gan’s group (58)
CDT	MnS@BSA	Size-controllable, biodegradable, and metastable γ-phase manganese sulfide nanotheranostics using BSA as a biological template for tumor pH-responsiveness traceable gas therapy-primed CDT	4T1 tumor-bearing mice	Peng Huang’s group (59)
CDT	GSH-Gated MnO_2_@PEI-IAA	For GSH-gated miRNA-21 signal amplification and GSH-activated MRI-guided CDT	MCF-7 tumor-bearing mice	Caina Xu and Huayu Tian’s group (60)
CDT	MCDION-Se	Nanoselenium-coated MCDION-Se for MRI guided CDT	HeLa and HK-2 tumor-bearing mice	Duohong Zou and Zhengyan Wu’s group (61)
RIT	^131^I-HSA-MnO_2_ NPs	Radionuclide ^131^I-labeled human serum albumin (HSA)-bound manganese dioxide nanoparticles (^131^I-HSA-MnO_2_) as a novel radioisotope therapy (RIT) nanomedicine platform for tumor microenvironment	4T1 tumor-bearing mice	Kai Yang and Zhuang Liu’s group (62)
Gene therapy	f-L-SQDs	The (f-L-SQDs)-folic acid-conjugated liposome core–shell co-doped Mn : ZnSe/ZnS/ZnMnS sandwiched quantum dots (SQD) to deliver cancer cell-targeted siRNA for dual-mode imaging (MRI and fluorescence imaging) and gene therapy	Panc-1 (ATCC CRL-1469)	Tze Chien Sum and Ken-Tye Yong’s group (63)
Photo-genetherapy	DNA/Mn NPs	A multifunctional theranostic nanoplatform-DNA/Mn NPs by encapsulating indocyanine green (ICG)-labeled CHA-DNAzyme prodrugs and MnO_2_ adjuvant into a biocompatible poly nanocarrier for photo-genetherapy strategy	MCF-7 tumor-bearing mice	Fuan Wang’s group (64)
Magnetic hyperthermia therapy	FIMO-NFs	Novel room-temperature FIMO-NFs to harness the advantages and potential of T1-T2 dual-mode MRI and magnetic hyperthermia therapy for precision medicine	U87MG tumor-bearing SCID mice	Jun Ding and Hai Ming Fan’s group (65)
PTT and CDT	PFN	A second near-infrared PFN for activatable MRI-guided synergetic PTT and CDT	Panc02 tumor-bearing mice	Ruizhi Wang, Yu Luo and Xiaolin Wang’s group (66)
photothermal-chemodynamic therapy	GNRs	A plasmonic modulation strategy of GNRs for imaging guided NIR-II photothermal-chemodynamic therapy	U87MG tumor-bearing mice	Peng Huang’s group (67)
photothermal-enhanced chemodynamic therapy	GSH-triggered Au@MnO_2_	An Au@MnO_2_ core–shell nanostructure as a GSH-triggered smart theranostic agent for PA and MRI-guided photothermal-enhanced chemodynamic therapy	4T1 tumor-bearing mice	Qiwei Tian and Shiping Yang’s group (68)
chemo-photodynamic therapy	CDM NPs	Oxygen-generating theranostic nanoparticles by hierarchically assembling DOX, Ce6, and MnO_2_ with poly-b-poly-b-poly for trimodal imaging-guided combined chemo-photodynamic therapy	MCF-7 tumor-bearing mice	ZhiYong Qian’s group (69)

PPT, photothermal therapy; MSOT, multispectral optoacoustic tomography; PA, photoacoustic; PB, Prussian Blue; FMCNPs, Fmoc-L-L/Mn^2+^/chlorin e6 nanoparticles; MnIO-dBSA, manganese doped iron oxide nanoparticles modified with denatured bovine serum albumin; HANP, hyaluronic acid nanoparticle; PDT, photodynamic therapy; SDT, sonodynamic therapy; DVDMS, organic sinoporphyrin sodium; CDT, chemodynamic therapy; MCDION-Se, nanoselenium coated manganese carbonate-deposited iron oxide nanoparticle; HSA, human serum albumin; RIT, radioisotope therapy; SQD, sandwiched quantum dots; ICG, indocyanine green; CHA, catalytic hairpin assembly; FIMO-NFs, ferromagnetic IMO nanoflowers; PFN, photothermal Fenton nanocatalyst; GNRs, gold nanorods; CDM NPs, chlorin e6-DOX-MnO_2_ nanoparticles.

### Imaging-Guided Photothermal Therapy

PTT, a combination of photothermal nanomaterials and light irradiation, becomes a clinically promising modality for cancers. It could controllably and selectively heat the target area to minimize thermal damage.

Many Mn-based hybrid nanomaterials used for imaging-guided PTT have been developed (11, 48, 71, 73–77), such as nanopetals of Mn_3_O_4_ hybrid nanomaterials for multifunctional imaging-guided PTT (51), a 2-D nanoplatform based on Cu_2_MnS_2_ nanoplates for MRI/multispectral optoacoustic tomography (MSOT) dual-modal imaging-guided PTT (52, 78), a plasmonic modulation strategy of Gold Nanorods (GNRs) through MnO_2_ coating for TME-responsive photoacoustic (PA)/MR duplex imaging guided NIR-II PTT (67), and a gold@ MnO_2_ (Au@MnO_2_) core–shell nanostructure as a GSH-triggered smart theranostic agent for PA and MR dual imaging-guided PTT (53, 68).

### Imaging-Guided Photodynamic Therapy

PDT has emerged as a promising therapeutic option for cancers, and it could generate cytotoxic oxygen-based molecular species *via* photosensitizer to ablate tumor growth by inducing cell apoptosis, necrosis, or autophagy. As a new noninvasive modality, PDT could enhance the conventional cancer treatment by overcoming drug resistance or escape pathways.

A lot of Mn-based hybrid nanoparticles were synthesized for imaging-guided PDT diagnosis and treatment (10, 54, 55, 79, 80). For example, Zhang et al. (10) have proven that Mn-doped iron oxide nanoparticles modified with denatured BSA (MnIO-dBSA) and Fmoc-L-L/Mn^2+^/Ce6 nanoparticles (FMCNPs) could improve antitumor PDT efficacy. Also, oxygen-generating theranostic nanoparticles (CDM NPs) with MnO_2_ could be applied for trimodal imaging-guided combined PDT in breast cancer (69). A multifunctional DNA-templated silver nanoclusters/porphyrin/MnO_2_ nanoplatform could be used for non-labeled fluorescence images of Zn^2+^ and 635-nm red light-triggered PDT (56). The MnO_2_ NP-based PDT nanocomplex could generate oxygen to overcome the limitation of insufficient oxygen level in tumors (55).

### Imaging-Guided Sonodynamic Therapy

SDT is an alternative promising method for cancers by generating reactive oxygen species (ROS), ROS to induce cell death with low-intensity ultrasound irradiation combined with nontoxic sonosensitizers (81, 82). It is characterized by high therapeutic efficiency with the advantages of noninvasiveness and mitigated side effects.

Mn-based theranostic agents could integrate imaging and therapy into a single nano-platform for imaging-guided SDT. It has been reported that even in the presence of skull, sinoporphyrin sodium (DVDMS) chelating with Mn (DVDMS-Mn-LPs) could effectively inhibit the tumor growth (57). The efficacy of SDT could be severely inhibited by hypoxia and high glutathione in TME, while a Mn porphyrin-based metal-organic framework (Mn-MOF) could improve antitumor immunity and immunosuppressive microenvironment upon ultrasound irradiation to show great potential for hypoxic cancer therapy (58).

### Other Imaging-Guided Therapies

Mn-based hybrid nanomaterials also hold great potential for many other traceable therapies for cancer, such as chemodynamic therapy (CDT) (60, 61), radiation therapy (83), magnetic hyperthermia therapy, and combination therapy (70, 84, 85).

For pH-responsive traceable gas therapy-primed CDT, a γ-phase Mn sulfide nanotheranostics using bovine serum albumin (MnS@BSA) could greatly suppress tumor growth (59). During radiation therapy, ionizing radiation will damage both normal tissues and tumors (86), and hypoxia within TME would often lead to the resistance to radiotherapy. To improve the effect of radiation therapy, radionuclide ^131^I-labeled human serum albumin (HSA)-bound MnO_2_ nanoparticles (^131^I-HSA-MnO_2_) could function as an effective agent to show great efficacy in tumor treatment (62). The novel room-temperature ferromagnetic wüstite iron-manganese oxide nanoflowers (FIMO-NFs) could harness the advantages and potential of dual-mode MRI and magnetic hyperthermia therapy to induce cancer cell apoptosis (65).

Mn^2+^-doped bio-response theranostic NP could be designed for tumor-specific enhanced combination therapy under the guidance of multimodal imaging (64, 66, 87, 88). Pd@Au bimetallic NP-decorated hollow mesoporous MnO_2_ (H-MnO_2_) NPs could achieve both nucleus-targeted PTT and TME hypoxia relief-enhanced PDT (89). As an intelligent nanoflower composite with multistage H_2_O_2_/pH/GSH-responsive properties, FHCPC@MnO_2_ could realize the specific release of drugs in tumor and significantly increase the synergetic therapeutic effect (72).

## Perspective

Cancer still remains a significant challenge worldwide, and the new discovered theranostic nanomaterials, such as Mn-based hybrid nanomaterials, which make diagnosis and treatment together in a unified platform, provide a novel therapy specialized for tumors. Since nanomaterials for theranostics create great new opportunities in developing CPM, this review focused on Mn-based nanoparticles with various applications (used as imaging agents, drug delivery, and theranostic agents) in CPM. Although a multitude of Mn-based hybrid nanomaterials have not been successfully used in the clinic, several well-designed Mn-based hybrid nanoparticles provide a new promising treatment option in the near future. What is worth emphasizing is that the novel nanoparticles should be thoroughly characterized, whether used as imaging agents, carriers for drugs, or theranostic platforms, and the toxicity studies in both cell culture and animal models are needed before they can be applied clinically. A future perspective is proposed for further research and development of complex targeted, multistage responsive nanomedical drug delivery systems with high intelligence, precision, and minimum toxicity for personalized cancer diagnosis and effective therapy. A major obstacle in designing theranostic Mn-based hybrid nanomaterials might be that providing target specificity to biomaterials for enhancing therapeutic effect and visualization in CPM. With the aid of multimode imaging, theranostic nanoparticles can visualize and monitor drug delivery and therapeutic responses at tumor site.

## Author Contributions

XL and PR contributed to the conception, design, writing, and final approval of the article. All authors contributed to the article and approved the submitted version.

## Funding

This work was supported by the National Natural Science Foundation of China (81771827, 82071986) and the 65th Batch of China Postdoctoral Science Foundation (2019M652806).

## Conflict of Interest

The authors declare that the research was conducted in the absence of any commercial or financial relationships that could be construed as a potential conflict of interest.

## Publisher’s Note

All claims expressed in this article are solely those of the authors and do not necessarily represent those of their affiliated organizations, or those of the publisher, the editors and the reviewers. Any product that may be evaluated in this article, or claim that may be made by its manufacturer, is not guaranteed or endorsed by the publisher.
